# Epidemiology and management of nitroimidazole-refractory giardiasis

**DOI:** 10.1093/jtm/taag002

**Published:** 2026-01-16

**Authors:** Anna Boté-Casamitjana, Rashmita Bodhani, Peter L Chiodini, Nicky Longley, Gauri Godbole, Sarah Eisen, Laura E Nabarro

**Affiliations:** Hospital for Tropical Diseases, University College London Hospitals NHS Trust, 235 Euston Road, London NW12BU, United Kingdom; Department of Clinical Parasitology, Health Services Laboratories, 1 Mabledon Pl, London WC1H 9AX, United Kingdom; Hospital for Tropical Diseases, University College London Hospitals NHS Trust, 235 Euston Road, London NW12BU, United Kingdom; Diagnostic Parasitology Laboratory, London School of Hygiene and Tropical Medicine, Keppel Street, London WC1E 7HT, United Kingdom; Hospital for Tropical Diseases, University College London Hospitals NHS Trust, 235 Euston Road, London NW12BU, United Kingdom; London School of Hygiene and Tropical Medicine, Keppel Street, London WC1E 7HT, United Kingdom; Hospital for Tropical Diseases, University College London Hospitals NHS Trust, 235 Euston Road, London NW12BU, United Kingdom; Department of Clinical Parasitology, Health Services Laboratories, 1 Mabledon Pl, London WC1H 9AX, United Kingdom; Hospital for Tropical Diseases, University College London Hospitals NHS Trust, 235 Euston Road, London NW12BU, United Kingdom; London School of Hygiene and Tropical Medicine, Keppel Street, London WC1E 7HT, United Kingdom; Children and Young People’s Division, University College London Hospitals NHS Foundation Trust, 235 Euston Road, London NW12B, United Kingdom; Institute of Child Health-Great Ormond Street, University College London, 30 Guilford St, London WC1N 1EH, United Kingdom; Hospital for Tropical Diseases, University College London Hospitals NHS Trust, 235 Euston Road, London NW12BU, United Kingdom; London School of Hygiene and Tropical Medicine, Keppel Street, London WC1E 7HT, United Kingdom

**Keywords:** Giardia duodenalis, giardiasis, Albendazole, India, mepacrine, nitroimidazole, refractory, resistance, travellers

## Abstract

**Background:**

*Giardia* is one of the most prevalent gastrointestinal protozoan pathogens globally. Nitroimidazole drugs are usually the first line of treatment, but nitroimidazole-refractory giardiasis (NRG), where infections fail to respond to these agents, is increasingly reported. At our centre, for laboratory-proven refractory giardiasis, a stepwise treatment ladder is used with albendazole plus tinidazole as second-line and mepacrine as third-line therapy.

**Methods:**

We conducted a retrospective review between 2020 and 2024 at the Hospital for Tropical Diseases, London. Laboratory records identified adults and children with *Giardia* detected by stool microscopy or molecular methods. Treatment details and clinical information were extracted from electronic patient records. NRG and clearance failure were defined as a positive molecular test at least two weeks after completion of treatment.

**Results:**

A total of 531 cases were identified. Of these, 59.0% (*N* = 311) were diagnosed through screening of high-risk individuals attending clinics for people seeking asylum and refugees (PSAR; Cohort A). The remaining 41.0% (*N* = 220) were symptomatic patients (Cohort B).

In Cohort A, the prevalence of NRG was 36.3% (49/135), with children showing significantly higher odds of clearance failure compared to adults (adjusted OR, 4.81; 95% CI, 1.09–21.40; *P* = 0.0367). In Cohort B, 34.1% of treatment-naïve travellers had NRG. Travel to India was strongly associated with NRG (adjusted OR, 4.19; 95% CI, 1.72–11.00; *P* = 0.002). Second-line treatment regimens with albendazole plus tinidazole were effective, and third-line mepacrine therapy was well tolerated with no adverse reactions and successful in nearly all cases.

**Conclusions:**

NRG was common in both asymptomatic and symptomatic individuals, particularly among children seeking asylum and travellers who had visited India. NRG likely results from multiple factors, including protozoal drug resistance and host-related factors. While our current treatment ladder appears effective, further research is needed to understand the mechanisms underlying treatment failure.

## Introduction

*Giardia* is a common cause of gastrointestinal infection worldwide, with personal and public health implications. It is transmitted via waterborne, foodborne and faecal–oral routes and is more prevalent in resource-limited settings due to inadequate sanitation. The prevalence is 2–5% in high-income countries and between 20% and 30% in low- and middle-income countries, especially among children and people living in rural areas.^1^

Acute giardiasis typically causes diarrhoea but can also result in malabsorption, flatulence and weight loss. Chronic symptoms may persist for months if the infection is not treated and may be associated with stunted growth in children in low- and middle-income countries.^2–5^ Both acute and chronic infections can be asymptomatic in some individuals.

Treatment is usually with 5-nitroimidazoles like metronidazole or tinidazole; however, nitroimidazole-refractory disease, known as nitroimidazole-refractory giardiasis (NRG), has increased over the past 10–15 years, with prevalence varying geographically.^6–12^

**Figure 1:**
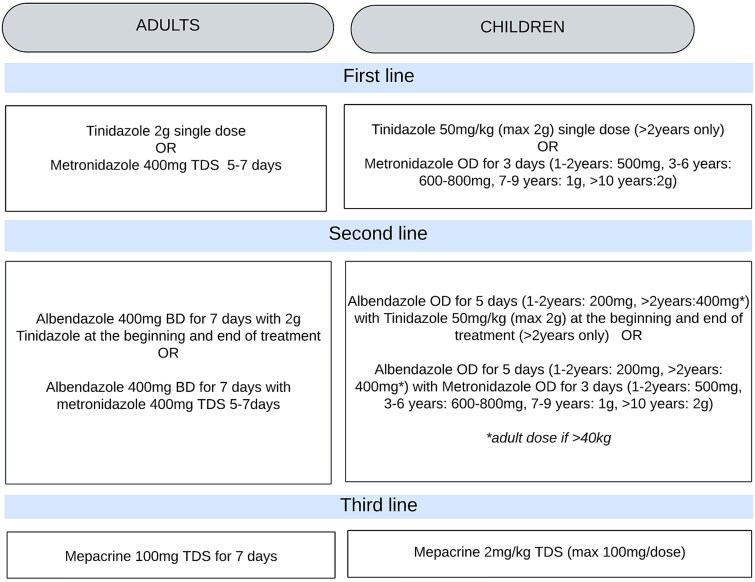
Treatment ladder. OD, once daily; BD, twice daily; TDS, three times a day.

The Hospital for Tropical Diseases (HTD) at University College London Hospitals (UCLH) NHS Foundation Trust is a specialist referral centre for infectious diseases, parasitology and refugee health. In the absence of susceptibility testing, a standardized empiric treatment ladder is used for patients who fail nitroimidazole therapy (Figure 1).

The primary objective of this audit was to evaluate the effectiveness of our treatment ladder in achieving laboratory-confirmed clearance in cases of NRG. The secondary objectives were to describe the epidemiology of giardiasis in our setting and to identify the characteristics and risk factors associated with NRG.

## Methods

We conducted a retrospective review of giardiasis over a 5-year period between 1 January 2020 and 31 December 2024 at the Hospital for Tropical Diseases, London. Laboratory records were interrogated to identify adults and children with giardiasis diagnosed by a single stool microscopy on a concentrated sample and/or molecular methods (in-house polymerase chain reaction).^13^ Patient demographics, travel history and clinical data were extracted from medical records. Clusters of infections were defined as households or groups of individuals presumed to have acquired the infection simultaneously.

At our centre, the standard first-line treatment for *Giardia* is a nitroimidazole agent, typically metronidazole or tinidazole. Patients with laboratory-proven clearance failure receive albendazole for 7 days combined with a single dose of tinidazole at both the start and end of therapy (commonly referred to as ‘sandwich’ therapy). If further failure occurs, third-line treatment is a 7-day course of oral mepacrine (Figure 1). This strategy is based on a previous audit conducted at our centre.^6^ In addition, the guideline recommends advising patients on infection control practices, investigating underlying conditions and assessing potential sources of reinfection.

For this study, NRG was defined as persistent detection of *Giardia* by stool microscopy and/or a polymerase Chain Reaction (PCR) at least 2 weeks after completion of treatment with metronidazole or tinidazole (treatment regimens, including dose and duration, are detailed in Figure 1). The available evidence demonstrates that *Giardia* DNA typically clears rapidly from the gut following effective therapy, with PCR tests becoming negative in nearly all patients within ~1 week after treatment completion.^14^ This supports the use of PCR as a sensitive tool for confirming microbiological cure.

For the analysis of NRG prevalence and treatment efficacy, only individuals with available post-treatment stool test results were included. In addition, cases considered by the treating clinician to represent reinfection or where the initial treatment was not tolerated were not classified as NRG cases.

We also assessed the relationship between clinical and parasitological outcomes by reviewing documented post-treatment symptoms. These were categorized as (i) resolved (complete symptom resolution), (ii) persistent (ongoing symptoms of similar severity) or (iii) residual (partial improvement reported by the patient but with ongoing mild symptoms such as burping, flatulence or bloating).

Data analysis was performed using Software for Statistics and Data Science (STATA, StataCorp, Texas, USA) version 17. The audit was registered according to local governance procedures. Ethical approval was not required.

## Results

### Demographics and populations identified

Between January 2020 and December 2024, 531 cases were identified. Of these, 47.5% (252/531) were aged under 18 years. The median age was 33.91 years (IQR 27.32–49.46) for adults and 11.94 years (IQR 6.61–16.36) for children.

Fifty-eight-point six percent (311/531) of patients were diagnosed after testing in an infection screening clinic for people seeking asylum and refugees (PSAR) (referred to hereafter as Cohort A). These patients were all asymptomatic. Of this cohort, 238/311 (76.5%) were children, and 72.9% (227/311) were male. The commonest countries of presumed *Giardia* acquisition were Afghanistan (72.5%, 221/305), Sudan (8.9%, 27/305) and Iran (4.3%, 13/305). No individuals in Cohort A were immunocompromised or living with human immunodeficiency virus (HIV).

The remaining 41.4% (220/531) cases (Cohort B) were diagnosed in treatment clinics, to which patients had been referred with symptoms. Ninety-three-point six percent (206/220) were adults, and 54.5% (120/220) were male. Travel history was documented in 95.0% (209/220). India was the most common country for presumed *Giardia* acquisition (26.8%; 56/209). Fifteen-point eight percent (33/209) of cases had no reported travel history and were presumed to have been acquired in the UK.

Cohort B was subdivided into two subgroups based on treatment history. Cohort B.1 consisted of treatment-naive individuals, the majority of whom were seen in a walk-in travellers’ clinic at the Hospital for Tropical Diseases. Cohort B.2 comprised individuals referred from community healthcare providers after at least one course of *Giardia* treatment and a persistently positive test. These cohorts had a comparable demographic profile, but overall, individuals referred from the community had a higher percentage of individuals returning from India and individuals who were immunocompromised (Table 1).

**Table 1:** Demographic characteristics of Cohorts A and B

Cohort	Total	Median age (IQR)	Sex (%)	Region of presumed acquisition (%)	Immunosuppressed (%)
**A**	311	15.47 years (8.08–17.92).	227 males (72.9) 84 females (27.0)	Asia 252 (81.0) Africa 36 (11.6) Europe 10 (3.2) Unknown 7 (2.3) Americas 6 (1.9)	0 (0)
**B**	220	36.65 years (28.53–53.5)	120 males (54.5) 100 females (45.5)	Asia 84 (38) Africa 50 (22.6) Europe 40 (18.1) Americas 30 (13.6) Unknown 16 (7.7)	27 (12.3)
B.1 (Treatment naïve)	165	36.3 years (28.4–53.3)	91 males (55.2) 74 females (44.8)	India 37 (22.4) UK 21 (12.7) Other 97 (58.8) Unknown 10 (6)	12 (7.2) 7 Chemotherapy 3 Immunosuppressants 1 HIV 1 Bone marrow transplant
B.2 (non-treatment naïve)	55	39.0 years (29.2–54.6)	29 (52.7) 26 (47.3)	India 19 (34.5) UK 12 (21.8) Other 23 (41.8) Unknown 1 (1.8)	10 (18.2) 2 Methotrexate 7 CVID 1 HTLV and steroids

### Diagnostic methods

Of the 531 confirmed cases, 98.1% (521/531) were PCR-positive on stool testing (Table 2). Among these, 42.4% (221/521) also had a positive microscopy result. Stool microscopy had an overall sensitivity of 47.0% compared to stool PCR (considered the gold-standard test). Notably, the sensitivity of microscopy compared to PCR was 39.0% in Cohort A, compared to 57.0% in Cohort B.

**Table 2:** Summary of diagnostic test results. The sensitivity of microscopy, using PCR as the reference standard, was calculated using the formula: Sensitivity = True Positives/True Positives + False Negatives

PCR	Microscopy			Total
	Positive	Negative	Not performed	
Positive	221	249	51	521
Negative	3			3
Not performed	7			7
Total	231	249	51	531

### Nitroimidazole refractory *Giardia* and treatment efficacy results

#### Cohort A: asymptomatic individuals diagnosed on routine screening

**Figure 2:**
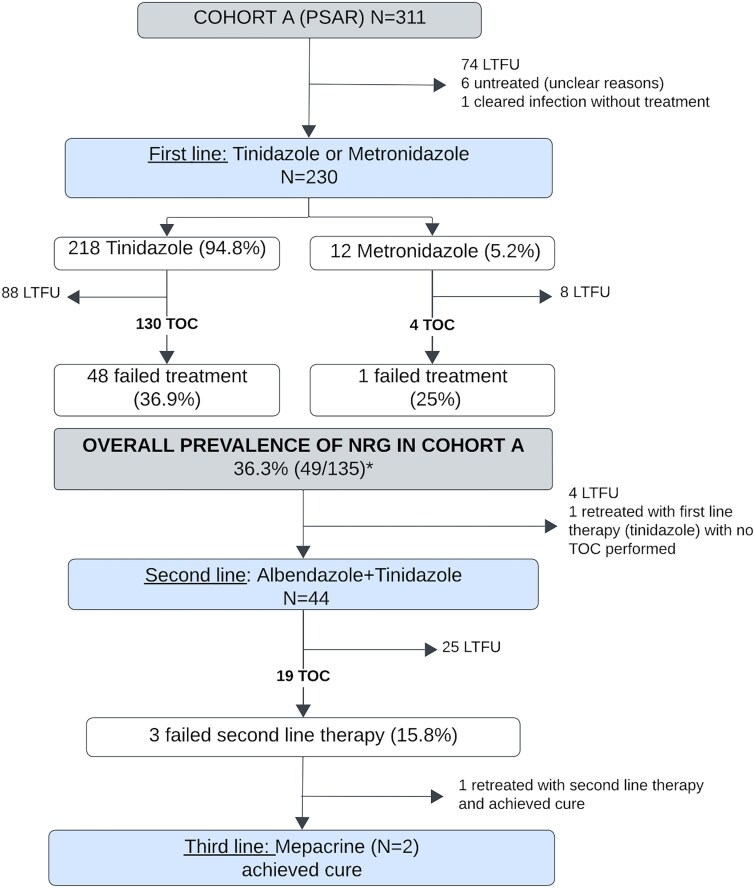
Flowchart of giardiasis cases, treatment outcomes and nitroimidazole-refractory giardiasis (NRG) prevalence in Cohort A. PSAR, people seeking asylum and refugees; LTFU, lost to follow-up; TOC, test of cure. ^*^Denominator includes the patient who was cured without treatment.

Among treated individuals in Cohort A, most received tinidazole (94.8%; 218/230) or metronidazole (5.2%; 12/230) as first-line therapy (Figure 2). Among this, a test of cure (TOC) was performed in 58.3% (134/230). One individual cleared the infection without treatment. Overall, 36.3% (49/135) individuals failed first-line treatment and met the criteria for NRG. Specific failure rates by drug are shown in Figure 2.

The majority (89.8%; 44/49) of patients diagnosed with NRG received second-line therapy with albendazole plus tinidazole. A TOC was available for 19 of these patients, among whom the failure rate was 15.8% (3/19). Mepacrine was given to two individuals who both achieved a cure.

In this cohort, no individuals were investigated for Immunoglobulin A (IgA) deficiency or coeliac disease.

Children under 18 had a significantly higher prevalence of NRG than adults: 40.0% (46/115) vs 13.6% (3/22). Age-stratified analysis showed that adults had lower odds of refractory giardiasis (unadjusted OR 0.24, 95% CI: 0.05–0.75; *P* = 0.027). After adjusting for sex, region of acquisition and being part of a cluster, adults remained less likely to experience clearance failure (adjusted OR 0.21, 95% CI: 0.03–0.84; *P* = 0.051). Region of infection acquisition was also an independent predictor: patients from outside Afghanistan had significantly lower odds of NRG (adjusted OR 0.09, 95% CI: 0.005–0.50; *P* = 0.025) than all other countries of acquisition. No independent association was found for sex or cluster status (Table 3).

**Table 3:** Univariate and multivariate analysis. mv, missing values. Risk factors: IgA, coeliac disease and HIV.

Variables	Risk factor	*n*	*n* (%) failure	Univariate			Multivariate		
				OR	95% CI	*P*	OR	95% CI	*P*
COHORT A									
Sex	Male	95	31	1			1		
	Female	42	18	1.55	0.73–3.267	0.25	1.30	0.58–2.95	0.52
Age (years)	<18	115	46	1			1		
	>=18	22	3	0.24	0.05–0.75	0.03	0.21	0.03–0.84	0.05
Travel (1mv)	Afghanistan	114	47	1			1		
	Other	22	1	0.07	0.01–0.34	0.01	0.09	0.01–0.50	0.03
Cluster	Yes	69	32	2.59	1.27–5.44	0.01	1.95	0.90–4.30	0.10
	No	68	17	1			1	1	
COHORT B									
Sex	Male	67	43	1	0.35–1.45	0.35	0.51	0.23–1.11	
	Female	70	40	0.71			1		0.10
Age (years)	<40	70	40	0.67	0.33–1.37	0.28	0.76	0.34–1.68	
	>40	67	43	1					0.48
Travel	India	47	36	3.00	1.37–6.96	0.01	4.19	1.72–11.00	
	Other	90	47	1			1	1	0.002
Cluster	Yes	33	21	0.87	0.33–1.97	0.74	1.22	0.47–3.20	
	No	104	62	1			1		0.69
Risk factors	Yes	5	3	1.06	0.17–8.24	0.94	0.68		
	No	132	80	1			1	0.09–6.03	0.70
Immunosuppression (11 mv)	Yes	18	13	0.50	0.15–1.44	0.22	0.33		
	No	108	61	1			1	0.09–1.05	0.07

#### Cohort B: symptomatic individuals referred to hospital clinics

Among Cohort B (*N* = 220), 165 patients were treatment-naïve at presentation (Cohort B.1). All were symptomatic, except for one individual, who was tested due to a positive result from a partner. The remaining 55 patients had been referred from the community with persistent symptoms despite prior treatment and tested positive for *Giardia* at our centre, meeting the criteria for NRG (Cohort B.2).

Overall, the most frequently reported symptoms in Cohort B were diarrhoea (171/219, 78.1%), abdominal pain (88/219, 40.2%), flatulence or bloating (70/219, 32.0%) and weight loss (43/219, 19.6%).

#### Cohort B.1. Treatment efficacy

**Figure 3:**
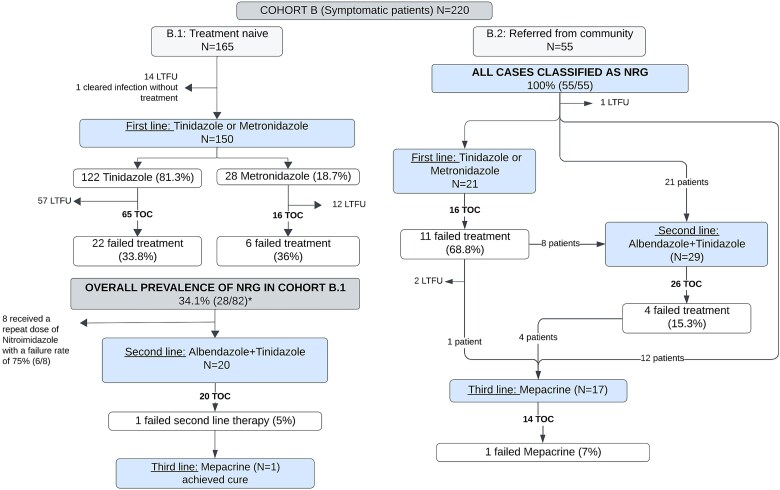
Flowchart of giardiasis cases, treatment outcomes and nitroimidazole-refractory giardiasis (NRG) prevalence in Cohort B.1 and B.2. ^*^Denominator includes the patient who was cured without treatment. Among Cohort B.2, one patient was treated with nitazoxanide for which TOC was not available. LTFU, lost to follow-up; TOC, test of cure.

Among those who received treatment, the majority were prescribed tinidazole (122/150; 81.3%), while a smaller proportion received metronidazole (28/150; 18.7%), and just over half of all treated patients (82/150; 54.7%) returned for a TOC. The overall prevalence of NRG in Cohort B.1 was 34.1% (28/82). Specific failure rates by drug are shown in Figure 3.

Seventy-one-point four percent (20/28) of patients with NRG were subsequently treated with albendazole plus tinidazole, and only one patient (5.0%; 1/20) failed this treatment regime subsequently requiring mepacrine and achieving parasitological cure. The remaining patients received a repeated dose of a nitroimidazole agent, which resulted in a high failure rate (75.0%; 6/8).

Among those with NRG, two patients (2/27; 7.4%) had a history of immunosuppression: one was receiving biological therapy for ankylosing spondylitis, while the other had diffuse large B-cell lymphoma and was undergoing chemotherapy. Data on immunosuppression were not documented for one individual. Screening for underlying conditions was variably performed: 25.0% (7/28) were tested for IgA deficiency, 42.9% (12/28) for coeliac disease and 96.4% (27/28) underwent HIV testing, all of which were negative.

#### Cohort B.2. Patients referred post-treatment from the community

Fifty-five cases of diagnosed *Giardia* were referred from the community to a specialist clinic. Eighty-three-point six percent (46/55) reported having received at least one treatment course with metronidazole, and 10.9% (6/55) had received tinidazole.

Twenty-one patients were retreated with metronidazole or tinidazole, of whom 16 underwent a TOC. Among these, 68.8% (11/16) experienced clearance failure. Second-line therapy had a failure rate of 15.3% (4/26), and one individual (7.0%; 1/14) failed with mepacrine, so they received a 1-month course of albendazole followed by paromomycin (750 mg TDS for 10 days) (Figure 3).

Screening for underlying conditions was variably performed in Cohort B.2. An IgA titre was performed in 45.0% (25/55) of cases, with two showing IgA deficiency. HIV serology was conducted in 78.0% (43/55), with one individual already known to be living with HIV. Coeliac serology was requested in 53.0% (29/55), and all results were negative. Finally, 18.2% (10/55) of individuals were immunocompromised. Seven were known to have Common Variable Immunodeficiency (CVID); three were on methotrexate [of which one was also known to have Human T-lymphotropic virus type 1 (HTLV-1)].

There were no instances of failure to tolerate medication in any of the cohorts, and more specifically, mepacrine was well tolerated with no reports of side effects.

#### Clearance failure and risk factors

In Cohort B, clearance failure was significantly more common among those who had recently travelled to India (unadjusted OR 3.00, 95% CI: 1.37–6.96, *P* = 0.01) than to other countries. This association remained significant and was stronger after adjusting for sex, age, cluster status, risk factors and immunosuppression (adjusted OR 4.19, 95% CI: 1.72–11.00, *P* = 0.002). No other variables were independently associated with clearance failure in this cohort (Table 3).

#### Correlation between symptoms and the test of cure

**Figure 4:**
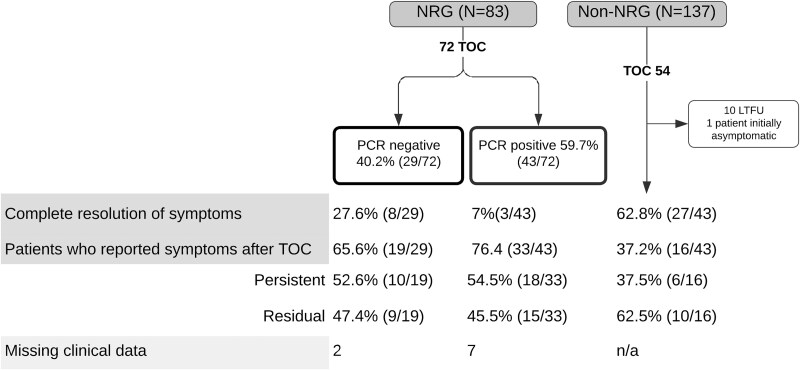
Correlation between symptoms and the test of cure in patients with and without nitroimidazole-refractory giardiasis (NRG). TOC, test of cure.

Among symptomatic patients who were treated for NRG (*N* = 83), most returned for a further test of cure after treatment (86.7%; 72/83). The median time between treatment and TOC was 36 days (IQR 20.0–52.3), and 62.5% (45/72) of patients had the test of cure performed at least a month after treatment (Figure 4).

Parasitological cure (PCR negative) was achieved in 40.2% (29/72) of patients. Of these, only 27.6% (8/29) reported complete resolution of their symptoms, and most (65.6%; 19/29) continued to experience some degree of gastrointestinal symptoms, either persisting with similar severity (52.6%; 10/19) or improving only partially with residual symptoms (47.4%; 9/19). On the other hand, 59.7% (43/72) of patients with NRG remained PCR-positive at TOC. Of these, a small proportion (7.0%, 3/43) were symptom-free following treatment, and most patients continued to experience symptoms (76.4%, 33/43). Within this group, 54.5% (18/33) continued to experience persistent symptoms, whereas 45.5% (15/33) had only residual symptoms.

Among patients with non-NRG that were followed up in clinic and had a TOC after initial treatment (*N* = 43), the majority experienced resolution of symptoms (62.8%; 27/43) (Figure 4). In those with ongoing symptoms, repeat OCP and PCR testing were performed to exclude a false-negative result. In this cohort, the median time between treatment and TOC was 40.5 days (IQR: 27.25–77).

## Discussion

### Epidemiology of giardiasis at our centre

We describe the epidemiology, risk factors and treatment outcomes of giardiasis in 531 patients during a 5-year period at our specialist centre. We observed a notably high prevalence of NRG in our cohort, with geographic and age-related differences in clearance failure rates.

Although we could not calculate the overall prevalence of giardiasis among PSAR attending our centre due to the absence of denominator data, our findings are consistent with previous studies reporting high infection rates among similar populations.^15–17^ A retrospective study performed in three London centres found a prevalence of *Giardia* of 7.7% in unaccompanied children seeking asylum.^16^ Similarly, a Swedish study found an overall rate of giardiasis of 1180/100 000 in PSAR, with the highest risk in persons from Afghanistan (3800/100 000).^15^

Similarly, *Giardia* remains a significant concern in India, with prevalence rates varying between studies, regions and populations.^18–20^ Our findings align with data from non-endemic countries, which show high giardiasis rates among travellers returning from Asia, particularly India.^21^^,^^22^ Public Health England (now the UK Health Security Agency, UKHSA) and the European Centre for Disease Prevention and Control identified India as the most common country of acquisition of giardiasis among travellers in its 2014 surveillance data.^23^^,^^24^

### Diagnostic methods

In our study, microscopy only detected 42% (218/518) of PCR-positive cases, and the sensitivity was notably lower in Cohort A. This may reflect lower parasite loads among asymptomatic or paucisymptomatic patients with chronic infection. It is also plausible that children may display different parasite shedding patterns than adults, perhaps making visual detection via microscopy more challenging.^25^ Lower sensitivity with microscopy compared to PCR has also been reported in other studies.^26–29^ The sensitivity of stool microscopy using a single stool sample is not adequate for diagnosing or confirming *Giardia* clearance, and molecular tests should be used where available.

### Prevalence and patterns of nitroimidazole-resistant giardiasis

In our study, most cases of NRG were observed in children from Cohort A (PSAR) and adult travellers returning from India (Cohort B). These findings point to distinct epidemiological and clinical patterns and will be discussed separately.

Within Cohort A, adults had significantly lower odds of refractory giardiasis compared to children. This association remained significant after adjusting for confounders, indicating that age is an independent risk factor for clearance failure. These results may reflect differences in host immunity and thus susceptibility to refractory infection, drug metabolism and absorption or behavioural factors that place children at higher risk of reinfection or inadequate treatment response than adults. Treatment doses were not directly observed in our cohort, but tolerability and compliance were interrogated in follow-up clinics.

Notably, the acquisition of giardiasis outside Afghanistan was independently associated with lower odds of clearance failure. This suggests that infections acquired in Afghanistan may carry a higher risk of resistance. However, most cases from Afghanistan occurred in children, and very few refractory outcomes were observed in adults. As such, the observed association with Afghanistan may partly reflect the higher burden of refractory cases among children, rather than a geographic effect *per se*. Specific data on *Giardia* resistance in Afghanistan are lacking; nevertheless, it is plausible that *Giardia* strains circulating may exhibit higher resistance rates, as this has been reported in neighbouring countries.^6–9^ The high prevalence of NRG among asymptomatic children is concerning, as they may act as reservoirs for household transmission, particularly in the crowded accommodations often experienced by these families. Additionally, the long-term consequences of chronic giardiasis in children living in non-endemic countries are unclear but warrant further investigation due to the potential impact on growth and development.

Among travellers (Cohort B.1), we found a prevalence of NRG of 34.1% (28/82). Our findings align with a previous study from our centre, which reported a rise in nitroimidazole failure rates from 15% in 2008 to 40% in 2013.^6^ They also align with international data, including a study from Cuba reporting a 54% failure rate with metronidazole,^30^ and studies in returning travellers and asymptomatic or mildly symptomatic children showing clearance failure rates around 20%.^7^^,^^31^^,^^32^

Travel history to India was strongly associated with an increased risk of NRG. These findings have also been reported in other cohorts of travellers returning from Asia and suggest that clearance failure may be driven by underlying drug resistance.^6–8^^,^^33^ In response to the strong association between NRG and travel to India observed in our cohort, our centre will now routinely offer ‘sandwich therapy’ (which is usually second-line treatment) as first-line treatment for patients returning from India, following shared decision-making with the patient.

When examining the relationship between TOC and clinical outcomes, ongoing symptoms were frequently observed in both patients who achieved microbiological clearance and those who remained PCR-positive. Distinguishing post-infectious gastrointestinal disorders such as transient lactose intolerance from active *Giardia* infection can be clinically challenging. In our experience, although many patients report clinical improvement after a first course of treatment, a significant proportion continue to experience chronic, non-specific symptoms. These individuals may be considered clinically cured in the absence of parasitological confirmation; however, persistent symptoms can still contribute to ongoing gastrointestinal and long-term morbidity.^35^^,^^36^ In our cohort, most TOCs were performed at least a month after treatment, suggesting that PCR positivity likely reflects persistent infection. Supporting this, a study from the Netherlands found that *Giardia* DNA is typically undetectable by PCR within a week of effective treatment, indicating rapid clearance and reinforcing that later PCR positivity represents ongoing infection rather than residual DNA.^14^

Despite some guidelines recommending against routine TOC in asymptomatic patients, persistent mild symptoms often complicate clinical assessment. In light of this and the higher rates of NRG reported in travellers from India and the risk of ongoing transmission within the community, we recommend a combined strategy of clinical evaluation and PCR-based follow-up to identify ongoing infection reliably.

### Efficacy of alternative treatment strategies and treatment ladder

Our second- and third-line treatment approaches resulted in low failure rates across the three cohorts (Table 4). Use of albendazole and tinidazole as second-line treatment achieved a cure rate of 84–95%. These findings align with previous studies that have used combinations of nitroimidazoles and benzimidazoles.^10^^,^^30^^,^^34^^,^^37^ A Cuban study, for example, reported an 87% cure rate with secnidazole plus mebendazole for NRG^30^ and, in a Norwegian study, a 79% cure rate with metronidazole combined with albendazole.^10^ Additionally, a small randomized controlled trial (RCT) in Italy demonstrated a 90% cure rate with albendazole and metronidazole.^37^

**Table 4:** A summary of the treatment efficacy in our centre. Denominators include only those with follow-up data available and a test of cure

Cohort	First line	Subgroup efficacy	Repeat course with first line	Second line	Third line
A	63.4% (85/134)	Adults 85.0% (17/20)	One individual retreated—unavailable outcome	84.2% (16/19)	100% (2/2)
		Children: 59.6% (68/114)			
B.1	65.5% (53/81)	India: 39.2% (11/28)	25% (2/8)	95.2% (20/21)	100% (1/1)
		Elsewhere:79.2% (42/53)			
B.2			31.2% (5/16)	84.6% (22/26)	92.8% (13/14)

Mepacrine demonstrated 100% efficacy in Cohort A and Cohort B.1, aligning with findings from several other studies.^6^^,^^30–32^ However, one individual in Cohort B.2 failed treatment with mepacrine. This subgroup had a higher proportion of individuals with immunosuppressive conditions and a history of travel to India. These factors may account for the observed differences in treatment outcomes. Despite the side effects of mepacrine having been reported in other studies, none of our patients, including children, required discontinuation or reported any adverse reactions. This finding contrasts with the results reported by Mørch *et al.*^10^

Finally, when a repeat course of first-line treatment was used following initial failure, efficacy was notably low across cohorts, with cure rates ranging from 25% to 37.5% where data were available, rendering this strategy suboptimal.

### Study limitations

Our data were collected from clinical notes and electronic health records, and their accuracy and completeness consequently varied. Secondly, although clinical notes were reviewed to detect cases of reinfection, incomplete documentation may have led to misclassification of cases. Thirdly, determining the country of initial infection was particularly challenging in Cohort A, as patients were often asymptomatic (making it difficult to establish the onset of symptoms), and some had travelled through multiple countries. Additionally, there is no universally accepted case definition of NRG. While many studies define it as treatment failure following a single full course, others require documented failure after two or more courses.^7^^,^^8^^,^^30^^,^^33^^,^^38^ This inconsistency complicates comparisons across studies and underscores the need for a standardized definition.

The interval at which patients should be tested following completion of treatment for *Giardia* infection is poorly defined and varies between studies. In our clinical practice, we perform a test of cure at least 2 weeks after treatment completion, based on available evidence suggesting that *Giardia* DNA typically clears from the gut within ~1 week in most patients.^14^ This interval is biologically plausible given that *Giardia* does not invade the bowel wall, and its DNA should therefore be rapidly eliminated in stool once the parasite is killed. Moreover, as intestinal epithelial cells are replaced approximately every 5 days, any residual adherent trophozoites would likely be cleared within this period. Nevertheless, uncertainty remains regarding the optimal timing of TOC. It is possible that PCR-based diagnostics may overestimate persistence of infection, whereas microscopy would significantly underestimate treatment failure due to its limited sensitivity.

Our analysis included only parasitologically confirmed cases of treatment failure. It is plausible that patients who were not tested—either because clinicians did not request it or because patients did not return a sample—were more likely to have achieved a microbiological cure, introducing selection bias and overestimating the treatment failure rates.

Finally, our limited evaluation of underlying immunosuppression means that host factors influencing treatment response could not be fully assessed. The retrospective design and relatively small sample size also constrained our ability to account for all potential confounders and to generalize findings beyond this setting.

## Conclusion

We report high rates of NRG across all cohorts, especially among children seeking asylum and travellers to India.

Despite the lack of international guidelines for NRG management, our treatment ladder demonstrated high efficacy and tolerability, achieving parasitological clearance in the majority of patients. The combination of tinidazole and albendazole was efficacious and therefore represents a reasonable second-line option. Mepacrine was an effective third-line option and well tolerated in our cohort.

Finally, the high prevalence of NRG in children underscores the need for increased clinical awareness and tailored treatments. Further studies are needed to identify host and pathogen factors driving treatment failure and guide targeted, evidence-based treatment.

## Data Availability

The data underlying this article will be shared on reasonable request to the corresponding author.
